# Association between overweight, obesity and self-perceived job insecurity in German employees

**DOI:** 10.1186/1471-2458-11-162

**Published:** 2011-03-14

**Authors:** Eva Muenster, Heiko Rueger, Elke Ochsmann, Stephan Letzel, André M Toschke

**Affiliations:** 1 Institute of Occupational, Social and Environmental Medicine, University Medical Center of the University of Mainz, Mainz, Germany; 2 Institute of Occupational and Social Medicine, University of Aachen, Medical Faculty, RWTH Aachen University, Aachen, Germany; 3 Department of Medical Informatics, Biometry and Epidemiology, University of Munich, Munich, Germany

## Abstract

**Background:**

Recent studies have shown an association between job insecurity and morbidity as well as mortality, however until now, knowledge about a potential association between job insecurity and overweight or obesity has been lacking.

**Methods:**

In order to identify a possible association between job insecurity and overweight or obesity, we analysed data from the German Socioeconomic Panel (GSOEP) 2004/2005, a longitudinal study of private households in Germany. In this representative cohort of the German adult population, living and working conditions were observed. Data on Body Mass Index (BMI) and self-perceived probability of job loss within the next 2 years were available for 10,747 adults either employed or attending training programs.

**Results:**

We identified 5,216 (49%) individuals as being overweight (BMI > 25 kg/m^2^) and 1,358(13%) individuals as being obese (BMI > 30 kg/m^2^). A total of 5,941 (55%) participants reported having concerns regarding job insecurity. In the multivariate analysis - after adjustment for relevant confounders - a statistically significant association between obesity and job insecurity (100% probability for losing the job in the following two years) could be observed with an adjusted odds ratio of 2.55 (95% confidence interval: 1.09-5.96).

**Conclusions:**

Because of these results, we were able to conclude that overweight and obese persons perceive job insecurity more often than their normal weight counterparts in Germany and that the concurrence of obesity and job insecurity might lead employees into a vicious cycle. Further research with an emphasis on the occupational setting might be necessary in order to establish useful preventive programmes at the workplace.

## Background

Being overweight and obese is often the result of "over-nourishment" and "too little physical activity", with an increasing prevalence in industrialized countries [1-10]. Both these factors are associated with co-morbidities such as high blood pressure, cardiovascular disease, gall bladder disease, insulin resistance leading to a manifest type 2 diabetes mellitus, and also some types of cancer [11-13].

Job insecurity is another risk factor that was reported to have negative health consequences such as mental disorders, high blood pressure and an increased vulnerability to infectious diseases [14-25]. When both factors - obesity and job insecurity - are found in one person, they might interact and lead to more serious outcomes - therefore we tried to take a closer look at the possible association between overweight/obesity and job insecurity.

In general, job insecurity can be investigated in view of two approaches: firstly by estimating job insecurity attributed to external factors and secondly by estimating self-perceived job insecurity [15]. Although at first glance the external approach seems to be more objective, the employee's actual perception of a secure or insecure work place was reported to be more important and predictive for subsequent poor health conditions [1].

To our knowledge there are few studies in which the association between body composition and job insecurity was examined [24,26,27]. Two of these included men only [26,27] and most studies examined weight development and changes in the Body Mass Index rather than focusing on how being overweight or obese could impact perceived job security.

The study presented here tries to close this gap of knowledge at least partially by proving the hypothesis that overweight or obese employees experience job insecurity more often and more intensely than their normal weight colleagues. This perceived job insecurity might induce a vicious cycle resulting in social, psychological, and health related problems which could have a negative impact on overweight or obese individuals' ability to perform adequately in their place of work.

## Methods

### Data sources and study population

In order to assess the association between overweight, obesity and perceived job insecurity we analyzed data from the German Socioeconomic Panel (GSOEP) 2004/2005. The GSOEP, which started in 1984, is an openly available longitudinal study of private households in Germany [28], and comprises a representative sample of the German general population older than 16 years [28,29]. Annual follow-ups (waves) are conducted. The 2004 wave included a total of 22,109 individuals in 11,796 households, while in 2005 a total of 21,105 individuals in 11,440 households were included. When combing the 2004 and 2005 waves of the GSOEP, longitudinal data on 20,120 individuals, aged 17 to 99 years and included in both data sets, were available for analysis. Further and more detailed information on the methodology of the GSOEP has already been published elsewhere [30,31].

Due to the usual age range of the working population in Germany (15-64 years) and due to the availability of information on weight, height and job insecurity the analysis conducted here was further confined to 10,747 apparently healthy employees and adults in training (17 to 64 years old). Data on weight and height as well as job related variables were gathered from the 2004 wave whereas self perceived job insecurity was exclusively documented in the 2005 wave.

### Body Mass Index

Self-reported weight and height were used to calculate the Body Mass Index (BMI; [kg/m^2^]). Overweight and obesity were defined according to the World Health Organisation (WHO) with overweight being defined by a BMI between 25.0 kg/m^2 ^and 29.9 kg/m^2 ^and obesity being defined by a BMI of 30.0 kg/m^2 ^or more [12].

### Biometric and social variables

Age was divided into four categories and put as a dummy variable in order to allow for non-linear relations. Marital status was stratified into four categories, 'single', 'married/cohabitant', 'divorced' and 'widowed'. Information on education was categorised in four groups, and ranged from "grammar school (13 years)", "secondary school (10 years), "compulsory school (9 years) to "no schooling grade"

### Job related variables

The assessment of job insecurity was based on objective and subjective variables. General information on occupational status was gathered in 2004 and included factors like the participant's current employment (y/n), whether he/she was employed in his/her trained job (y/n), a rating of the job market for similar posts and information on the sector of the current job (private or public sector).

Occupational classifications were assigned on the basis of the respondent's current or previous job title and their managerial/supervisory responsibility. The Erikson-Goldthorpe-Portocarero (EGP) class schema was used. It consists of 11 categories characterising labour market conditions in a non-hierarchical way [32]. The EGP classes were reported to differentiate between amount and pace of work possibly representing the decision latitude or work environment control according to Karasek [33].

In 2005 the specific items "job insecurity" and "occupational changes within the last 12 months" were obtained. These concerned objective information such as a current job searching status at a job centre (searching vs. not searching for new jobs), current occupational status, job changes after 2003 and sickness leaves in 2004 as well as subjective information like self rating questions on the employee's satisfaction with his post, on job guarantee for the next 2 years, upcoming job degradation and on adequate salary according to post.

Job insecurity was specifically queried by following question: "How likely is it that the following career changes will take place in your life within the next two years?...lose your job?" The respondents estimated the probability of such a change according to a scale from 0 to 100; while 0 means that such a change will definitely not take place and 100 means that such a change definitely will take place. A-priori categories "0%", 10-30%", "40-60%", "70-90%" and "100%" were generated.

### Statistical analysis

Odds ratios for overweight/obesity and job insecurity were calculated with corresponding 95% confidence intervals based on a binomial distribution [34]. Multivariate analyses were performed using binary logistic models with categorical explanatory variables as dummy variables which are represented in Table 1 and Table 2. Additional age and sex-stratification of the data was performed. We used a backward selection to identify the final model estimating the adjusted odds ratios. The random error level was set to alpha = 0.05.

**Table 1:** Socio-demographic description of the study population (n = 10,747), stratified for overweight and obese people

		total	overweight^a^		obesity^b^	
		N	N	%	N	%
Total		10747	5216		1358	
Sex	female	4951	1790	36.2	556	11.2
	male	5796	3426	59.1	802	13.8
	p-value		< 0.001		< 0.001	
Age	17-29 years	1803	473	26.2	107	5.9
	30-39 years	2754	1236	44.9	292	10.6
	40-49 years	3384	1773	52.4	474	14.0
	50-64 years	2806	1734	61.8	485	17.3
	p-value		< 0.001		< 0.001	
marital status	married/cohabitant	6984	3808	54.5	1008	14.4
	single	2733	881	32.2	211	7.7
	divorced	893	437	48.9	109	12.2
	widowed	137	90	65.7	30	21.9
	p-value		< 0.001		< 0.001	
nationality	German	9975	4794	48.1	1253	12.6
	non-German	772	422	54.7	105	13.6
	p-value		< 0.001		0.062	
educational level	grammar school (13 years)	3265	1368	41.9	288	8.8
	secondary school (10 years)	3768	1767	46.9	469	12.4
	compulsory school (9 years)	2655	1557	58.6	466	17.6
	no schooling grade	184	100	54.3	31	16.8
	other	631	338	53.6	83	13.2
	not specified	244	86	35.2	21	8.6
	p-value		< 0.001		< 0.001	

^a ^Overweight is defined by a BMI between 25 kg/m^2 ^and 29.9 kg/m^2^. ^b ^Obesity is defined by a BMI of 30 kg/m^2 ^or more.

**Table 2:** Employment characteristics of overweight and obese persons

		total	overweight^a^		obesity^b^	
		N	N	%	N	%
working hours	full-time	7647	4092	53.5	1031	13.5
	part-time	1907	723	37.9	211	11.1
	apprenticeship	466	120	25.8	29	6.2
	minor employment	727	281	38.7	87	12.0
	p-value		< 0.001		< 0.001	
duration of employment contract	unlimited	7774	3893	50.1	1026	13.2
	temporary	1223	446	36.5	102	8.3
	self-employed	1750	877	50.1	230	13.1
	p-value		< 0.001		< 0.001	
occupational categories	upper grade of civil service	1335	671	50.3	133	10.0
	lower grade of civil service	2464	1067	43.3	287	11.6
	routine clerical/sales	1108	425	38.4	123	11.1
	routine service-sales	1222	524	42.9	133	10.9
	small employers	521	292	56.0	69	13.2
	independent	518	241	46.5	66	12.7
	skilled manual jobs	1699	959	56.4	226	13.3
	semi-unskilled manual	1731	956	55.2	303	17.5
	farm labour	149	81	54.4	18	12.1
	p-value		< 0.001		< 0.001	

^a ^Overweight is defined by a BMI between 25 kg/m^2 ^and 29.9 kg/m^2^.^b ^Obesity is defined by a BMI of 30 kg/m^2 ^or more.

All calculations were carried out with the statistical software package SPSS 13.0 (SPSS Inc., Chicago, IL, USA).

## Results

### Descriptive analysis

The data of 4,951 (46%) female and 5,796 (54%) male employees was analysed. It was found that 5,216 (49%) employees were overweight and 1,358 (13%) employees obese according to the WHO classification. A total of 5,941 (55%) participants experienced job insecurity to some extent (i. e. a fear of job loss in the following 24 months of more than 0%); 2,593 (24%) of them estimated the probability of losing their job within the next 24 months to be higher than 40%, which is regarded as high job insecurity.

Table 1 depicts the demographic characteristics of the participating overweight and obese employees. Major differences between normal and overweight/obese persons were found with regards to sex, age, marital status and educational level. For male subjects, older age, being married or widowed and lower educational level were significantly associated with a higher prevalence of overweight and obesity. Migration background (not having the German citizenship) was significantly associated with being overweight but not with being obese (Table 1).

Apart from that, significant associations between occupational variables and overweight or obesity became obvious. For example, full-time employees were more often overweight compared to part-time employees. Employees with temporary contracts showed a lower prevalence of being overweight and of obesity. Additionally, being overweight or obese seemed to be associated with the EGP classification of occupation (Table 2).

### Multivariate analysis

While the unadjusted analyses showed no association between job insecurity and being overweight or obese (data not shown), adjustment for age, sex, marital status, nationality, education, occupational class, contract type (temporary vs. permanent) and other confounders in the multivariate model led to significant results in the association between obesity and high job insecurity (aOR: 2.55; 95% CI: 1.09-5.96) (Table 3). Also, the adjusted model hinted at a exposure-response relationship regarding self-rated job insecurity and overweight and obesity.

**Table 3:** Self-reported job insecurity and risk of overweight and obesity - adjusted end model

		overweight		obesity	
		**OR^a^**	**95% CI^b^**	**OR^a^**	**95% CI^b^**

job insecurity	0%	1.00		1.00	
	10-30%	1.08	[0.97, 1.19]	1.44	[0.82, 2.51]
	40-60%	1.11	[0.99, 1.25]	1.12	[0.59, 2.13]
	70-90%	1.16	[0.94, 1.43]	1.86	[0.83, 4.16]
	100%	1.47	[1.09, 1.99]	2.55	[1.09, 5.96]

^a ^odds ratio (OR) and ^b ^95%- Confidence interval (95% CI) from multiple unconditional logistic regression adjusted for sex, age-group, marital status, nationality, educational level, occupational categories, working hours, duration of employment contract

Due to the obviously relevant interaction between variables job insecurity and age, as well as sex, we additionally stratified the multivariate model of obesity by age and sex. Significant results are shown in Table 4. Job insecurity was directly related to obesity in employees younger than 30 years. Also, an influence of self perceived job insecurity of 70% and more on the prevalence of obesity was found amongst men and women older than 50 years (Table 4).

**Table 4:** Self-reported job insecurity and risk of obesity - total and stratified analysis for age and sex, results of significant associations in the adjusted end model

		< 30 years, both sexes		> 50 years, female		> 50 years, male	
		OR^a^	95% CI^b^	OR^a^	95% CI^b^	OR^a^	95% CI^b^
job insecurity	0%						
	10-30%	1.55	[0.88, 2.73]	0.81	[0.51, 1.30]	1.35	[0.92, 1.98]
	40-60%	1.29	[0.67, 2.48]	0.70	[0.40, 1.20]	0.84	[0.51, 1.38]
	70-90%	1.93	[0.85, 4.38]	1.29	[0.52, 3.20]	2.94	[1.22, 7.10]
	100%	2.96	[1.25, 7.00]	1.73	[0.49, 6.17]	13.84	[1.69, 113.44]

^a ^odds ratio (OR) and ^b ^95%- Confidence interval (95% CI) from multiple unconditional logistic regression adjusted for marital status, nationality, educational level, occupational categories, working hours, duration of employment contract

## Discussion

After adjustment for relevant socioeconomic confounders, a direct association between overweight and obesity and self-perceived job insecurity could be observed with a focus on employees younger than 30 years or older than 50 years. This outcome is of relevance for activities in occupational and public health, since it supports the possibility of a link between overweight/obesity and the prevalence of self-experienced job insecurity. This link might explain the current development of a rapid rise in both entities [6] on a psychosocial basis. It also stresses the importance of employers getting involved in the struggle against the obesity epidemic [35,36].

To our knowledge this is the first study examining this association as such, though other studies were already conducted to explore an association between job insecurity or job strain and weight or BMI-changes [20,22,24,26,37]. These studies reported work sector dependent results. While, for example the manual workers of the Michigan Study [38] showed no alterations of their BMI shortly before the factory shut-down, the civil servants of the Whitehall-II-Study [22,24] had a higher BMI shortly before privatisation. Apart from that, in the Whitehall-II-Study as well as in our analyses being female was inversely related to obesity. These findings suggest a gender- and occupation-specific effect of job insecurity on BMI, and consequently on overweight and obesity.

Job insecurity is a major determinant of a psychological "burden", and probably similar to that perceived from job strain. Job strain was reported to be associated with obesity in previous cross-sectional studies [39-41], two of which reported an association between job strain and central obesity in two male cohorts [40,41], while no association could be observed in females or in the general study population.

Our findings should be discussed, keeping in mind the results of the other studies which suggested a cause-effect relationship between job strain and chronic diseases involving weight gain and/or increased (central) obesity [37,42,43]. In order to differentiate more accurately between job strain caused by the job itself and job strain caused by economic circumstances (which might in turn increase the perception of job insecurity), a more detailed analysis of job strain and/or self-perceived job insecurity is needed in future studies in order to enable the development of more focussed public health activities and occupational preventive measures. The consideration of self-perceived probability of job loss is of major importance at any rate, because it might be the more potent stressor compared to general job strain [18].

Job insecurity could be a major source of stress and, apart from being a stressor in itself, it might also affect job satisfaction as well as, in the long term, salary, future plans and future quality of life. It might lead an employee into some sort of vicious cycle. In the beginning, self-perceived job insecurity can have direct effects on motivation and on quality of work [44]. Low quality of work and low motivation might decrease work efficiency and this might lead to a change in the employer's behaviour towards his employee, resulting in an increase in the self-perceived job insecurity of the employee. Being overweight or obese might lead into this vicious cycle. On the one hand, job insecurity might alter the eating behaviour, so that employees might become obese. Poor stress management based on the strain of real or perceived job insecurity is another factor which can contribute to non-health-conscious behaviour such as „increased food intake leading to obesity"[45]. In the event of job insecurity, eating can become a compensation and gratification. Obese employees, on the other hand, might have a higher likelihood of being released due to the social stigma of being overweight or obese or due to the well-known increased risk of co-morbidities, such as high blood pressure and cardiovascular disease and the resulting increased time of absence from the work place [46,47]. Therefore obesity might have an additive effect on the perception of job insecurity.

But apart from these associations, job insecurity might also influence an employee's social network [48,49]. This result might have further impact on the development of obesity which is often found to be associated with certain family and/or social patterns.

Although we are reporting data from a longitudinal cohort study, the BMI data and job insecurity data were obtained nearly at the same time (1 year difference from 2004 and 2005). In general, we cannot rule out reverse causality between BMI status and job insecurity, but the latter measured in 2005 cannot be the cause for the former measured one year before. However, both directions of the association have implications for overweight and obese persons, either with job insecurity as an additional cause or as an additional condition triggering other co-morbidities.

Height and weight were self-reported and could have been biased in order to give socially acceptable answers. This might result in reporting bias. A potential non-differential misclassification cannot be ruled out and might have attenuated the association between job insecurity and overweight or obesity. A differential misclassification might result in different prevalence rates for being overweight or obese. However, this is similar to a change of the cut-off values for overweight or obesity and it has been shown that a change in cut-off values still allows the assessment of relationships [50]

The information on job insecurity was self rated and might differ from objective measures such as dismissals or appraisals. A potential reporting bias might mirror a pessimistic approach to life and hence accentuate negative feelings about situations [51]. However, subjective measures are more likely to reflect the individual's psychological situation, which is of major importance for related co-morbidities as compared to objective variables which might not reflect the self perception. This might also be the reason for negative findings in studies examining objective measures of job insecurity and weight tracking [38].

## Conclusions

The association between job insecurity and being overweight as well as being obese was moderate, although our data suggest a high public health impact due to the high prevalence of job insecurity and the emerging obesity epidemic. Because of the significant risks to the population even moderate associations can be of a high public health impact when coinciding with high prevalence rates of exposure. Job insecurity might represent an important psychological burden which again might trigger obesity and other diseases. Our results highlight the need of an occupational risk assessment which includes triggers for psychological stress in general but which can also especially focus on self-perceived job insecurity. As employees spend a large amount of their lifetime on their job and as a safe job is important for making a living, the fear of losing one's job might be an especially potent stressor. Additionally, we concluded that when speaking of the risks of being overweight and obese, not only the individuals' nutritional habits or their level of physical activity should be included, but also occupational status and occupational situation. It seems to be necessary that stress factors like job insecurity should be included in prevention strategies for overweight and obese employees; furthermore, the social stigmatisation of being overweight and obese has to be combated.

## Competing interests

The authors declare that they have no competing interests.

## Authors' contributions

Design of the study: EM, AMT, SL. Data analysis: EM, AMT. Writing of manuscript: EM, AMT, HR, EO. All authors read and approved the final manuscript.

## Pre-publication history

The pre-publication history for this paper can be accessed here:

http://www.biomedcentral.com/1471-2458/11/162/prepub
